# Influenceable and Avoidable Risk Factors for Systemic Air Embolism due to Percutaneous CT-Guided Lung Biopsy: Patient Positioning and Coaxial Biopsy Technique—Case Report, Systematic Literature Review, and a Technical Note

**DOI:** 10.1155/2014/349062

**Published:** 2014-11-10

**Authors:** Gernot Rott, Frieder Boecker

**Affiliations:** Department of Radiology, Bethesda Hospital Duisburg, Heerstraße 219, 47053 Duisburg, Germany

## Abstract

Following the first case of a systemic air embolism due to percutaneous CT-guided lung biopsy in our clinic we analysed the literature regarding this matter in view of influenceable or avoidable risk factors. A systematic review of literature reporting cases of systemic air embolism due to CT-guided lung biopsy was performed to find out whether prone positioning might be a risk factor regarding this issue. In addition, a technical note concerning coaxial biopsy practice is presented. Prone position seems to have relevance for the development and/or clinical manifestation of air embolism due to CT-guided lung biopsy and should be considered a risk factor, at least as far as lesions in the lower parts of the lung are concerned. Biopsies of small or cavitary lesions in coaxial technique should be performed using a hemostatic valve.

## 1. Introduction

Percutaneous computed tomography- (CT-) guided lung biopsy, an everyday practice in many institutions, has well-known potential complications, in numbers, mainly occurring as pneumothorax and pulmonary bleeding with both of them normally requiring little or no further treatment. Systemic air embolism is a feared and potentially fatal complication but with very low reported incidences ranging from 0,001% to 0,003% according to publications dealing with greater series of biopsies [1, 2]. Statistically, most radiologists performing percutaneous lung biopsies will never have to deal with this complication. On the other hand one study with a smaller patient population recently reported an incidence of 3,8% [3].

Risk factors for systemic air embolism have been speculated, postulated, and reported; these include use of a coaxial biopsy system, number of biopsies, needle path through a longer distance of ventilated lung, coughing during the procedure, positive pressure ventilation, location of lesion in the lower lobes or lower parts of the lung, location of the lesion above the level of the left atrium, vasculitis, and small or cavitary lesions with some of these being influenceable or even avoidable and others not [2–9].

Prone positioning as a truly influenceable factor has been considered a risk factor [3] but to our knowledge has never been evaluated systematically in a literature review.

Our very first case of systemic air embolism after CT-guided lung biopsy occurred at our institution after performing the procedure for much more than 10 years with a frequency of at least 50 cases per year. We are presenting this case, as we strongly believe that, in the light of the very low incidence of this complication, every single case should be published in detail in a medical journal.

The serious complication led us to consider whether there might be possibilities to improve safety of percutaneous lung biopsy in this regard. We investigated the factor of patient positioning during lung biopsy in form of a literature review and came across a technical solution to eliminate one main mechanism for air embolism, which is presented in an additional technical note.

## 2. Material and Methods

We performed a literature search in PubMed in May 2014 for reported cases of systemic air embolism due to CT-guided lung biopsy with regard to the subject patient positioning during biopsy using the search terms “lung,” “biopsy,” “air,” and “embolism.” Abstracts were read and all articles of potential relevance were read in full, if furthermore necessary and available for free. In addition the reference lists of identified articles were checked to identify further relevant articles. Articles published in English, German, and French were selected. “Systemic air embolism” was defined as evidence of air within the left heart or greater circulation proven on CT-images. “Due to CT-guided lung biopsy” was defined as during, immediately after, or at least in a clearly temporal coincidence with CT-guided lung biopsy. Excluded were cases of air embolism due to conventional fluoroscopic-guided lung biopsy, due to needle marking of lung lesions and due to radiofrequency ablation of the lung.

## 3. Case Report

A 57-year-old man with a large cavity of the right upper lobe of the lung was referred to our institution for further diagnostic, after a diagnosis had not been able to be made during a hospital stay in another clinic. Several noninvasive diagnostics for pulmonary tuberculosis were negative. A flexible bronchoscopy with bronchial wash cytology, an aspiration cytology, and an endobronchial forceps biopsy were performed and also revealed neither tuberculosis nor tumour.

**Figure 1:**
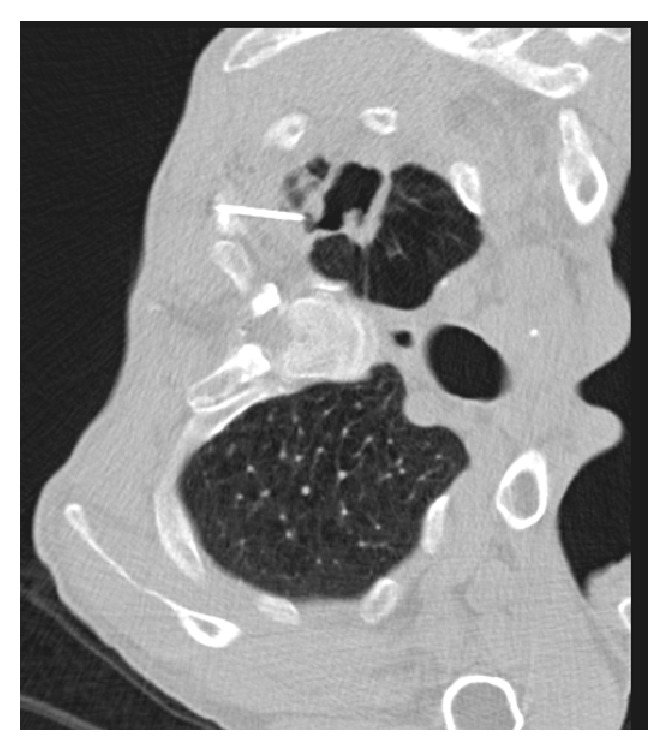
CT image obtained during lung biopsy with the patient in left lateral position and the tip of the guiding needle in the wall of a large cavitary lesion of the right upper lobe.

**Figure 2:**
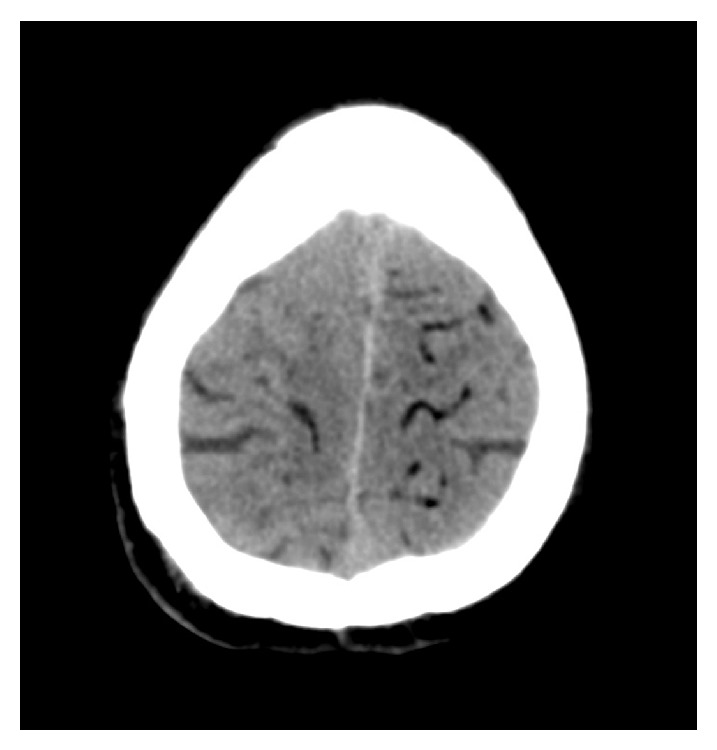
CT image of the brain after lung biopsy with signs of cerebral air embolism, typically visible as subcortical serpentiform formations with negative Hounsfield units.

A percutaneous lung biopsy was requested. For this purpose, the patient was positioned left-lateral, that is, on the contra-lateral side, and a percutaneous biopsy under CT guidance was performed using a core needle biopsy system with a 17/18-gauge sized coaxial needle (Bard Magnum Biopsy System, Bard TruGuide Disposable Coaxial Needle; C. R. Bard, Tempe, USA). The guiding needle was placed 17 mm intrapulmonarily in the dorsal cavity wall and four cutting biopsies in slightly different directions were performed, each with a penetration depth of 22 mm (Figure 1). During these procedures, a one-time coughing episode of the otherwise clinically unremarkable patient was noticed. After the biopsies control-scans were performed, as it was routinely done in our institution until the described case, that is, with only a few selected scans in the area of the lesion and the midfield of the lungs to rule out pneumothorax or relevant bleeding, failing to detect any complication. After this the patient was turned back into the supine position and put into his bed, where he sat up. This manoeuvre immediately made him become unconscious. A promptly performed cranial CT scan showed a cerebral air embolism (Figure 2). After being intubated and ventilated in a Trendelenburg position, as far as was practically possible on the CT table, a control-scan of the entire thorax was performed to rule out air in the left heart or ascending aorta.

The patient was monitored in the intensive care unit and within few hours transferred to another facility where he received hyperbaric oxygen therapy. After five days, he was transferred back to our hospital. Follow-up cranial CT showed no signs of ischemia or cerebral infarction. Subjectively the patient became completely free of complaints, but neurological examination showed signs of a minor neurological deficit in the form of a pyramidal tract syndrome on the right side.

Lung biopsy specimens showed an inflammatory disorder but did not indicate presence of a tumour. The patient was dismissed 18 days after the incident.

Recapitulating this case, at least four risk factors for air embolism due to CT-guided lung biopsy can be pointed out: a cavitary lesion, coughing during the procedure, location of the lesion above the level of the left atrium, and use of a coaxial needle in the conventional manner, that is, with opening the outer cannula to the atmosphere several times.

## 4. Results

### 4.1. Literature Review

Our literature search for reported cases of systemic air embolism due to CT-guided lung biopsy identified a total of 46 publications, which met our criteria as mentioned above, published between March 1988 and May 2014. Of these, one paper, whose results are included in another and four other papers, in which patient positioning is not mentioned or visible at all, were excluded, resulting in 41 publications [2–6, 10–45]. With our own case report included, a total of 42 papers were analysed. The number of cases reported per publication ranged from one to twenty-three with thirty-six 1-case reports, one 2-cases report, one 3-cases report, two 4-cases reports, one 10-cases report and one 23-cases report. In total, there were 82 case reports, but in 5 cases patient positioning was not precisely mentioned in a sufficient manner, so that 77 cases remained in the final analysis.

From the total of 77 cases of air embolism due to CT-guided lung biopsy 47 (61.0%) were performed in a prone position, 20 (26.0%) in a supine position, 3 (3.9%) in a right-lateral position, 6 (7.8%) in a left-lateral position and 1 (1.3%) in a lateral, but not otherwise specified position.

Data are illustrated and summarised in Table 1.

As our literature review investigated the technical aspect of patient positioning during biopsy, it is not surprising that the selected literature gives relatively imprecise information about other aspects of biopsy technique. These are briefly summarised in the following.

With respect to biopsy type 61 cases (74%) were performed as core biopsy, 9 cases (11%) as aspiration biopsy, and 4 cases (5%) as aspiration and core biopsy together (8 cases without precise information in this respect). As related to type of needle used 24 cases (29%) were performed as single-needle biopsy and 56 cases (68%) as coaxial biopsy (2 cases with no or imprecise information in this respect). Accurate information regarding needle size was available in 63 cases, with the most commonly used size in single-cannula technique being 18 gauge (12 cases), and in coaxial technique a needle combination of size 17/18 gauge (28 cases). Information concerning the number of conducted biopsies per case were available in 69 cases with more or less precise data. In the paper with by far the most noted cases [3], namely 23, the number of biopsies is described not entirely accurate as, “it was attempted to obtain at least 3 contiguous tissue cylinders”. So in most cases the number of conducted biopsies would be “at least 3”. Reviewing only the data naming the exact number of biopsies taken, in most cases (20) only one biopsy had been conducted.

### 4.2. Technical Note

Concerning the abovementioned potential risk factors for air embolism, coaxial biopsy technique has been the topic of commentaries in many papers but in our view thus far has surprisingly not received sufficient attention. Heretofore published strategies for preventing the exposure of the outer cannula of a coaxial biopsy needle to the atmosphere by removal of the internal stylet during lung biopsy include immediate occlusion of the guiding cannula with a saline drop, with the inner stylet, with a cap, or with the finger [46]. Even when valuable advice is given as “when performing the coaxial technique, never leave the outer cannula inside the patient without the inner stylet” [47], it is well-meaning, but technically unfeasible.

In coaxial biopsy technique, the outer cannula is opened to the atmosphere at least for a fraction of a second, when the mandrin is removed and each time the inner stylet is inserted into or removed out of the outer cannula. This means that a biopsy in usual coaxial technique with acquisition of, for example, three specimens, opens the outer cannula six times to the atmosphere, which in everyday practice probably adds up to a few seconds. For biopsies of relatively large lesions, where the guiding needle can be placed safely within the lesion, this is irrelevant, but of course things look quite different for small or cavitary lesions.

**Figure 3:**
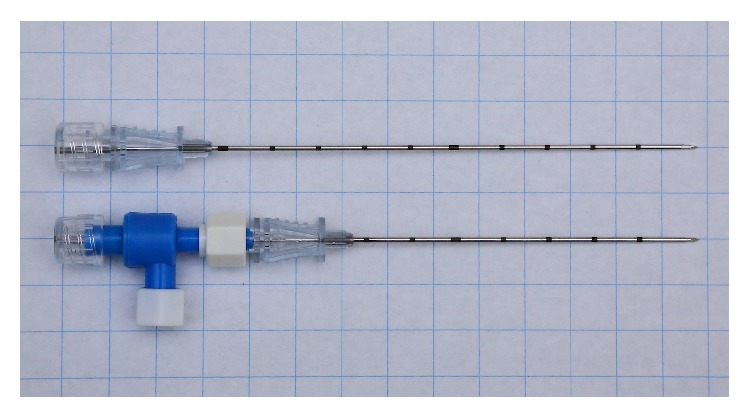
Combination of a coaxial biopsy guiding needle and a hemostatic valve for percutaneous lung biopsy. The 10 cm guiding needle connected with the hemostatic valve and closed with the inner cannula of the 13 cm device measures as much and can be handled according to the corresponding 13 cm device.

At least theoretically, this problem can be resolved with use of a hemostatic valve. A coaxial biopsy needle equipped with a hemostatic valve is not available in the open market, so it has to be assembled from separate existing components. The hemostatic valve connected should be short, lightweight, and, if possible, without additional components such as y-connector, connecting tube, or stopcock. For biopsy, we use a reusable core biopsy instrument and a disposable coaxial biopsy needle (Bard Magnum Biopsy System, Bard TruGuide Disposable Coaxial Needle; C. R. Bard, Tempe, USA). The guiding needle is available in four different lengths of 7 cm, 10 cm, 13 cm, and 17 cm, each one equipped with a flexible slip ring style depth stop for adjusting placement as necessary. For the hemostatic valve we use a simple selfadjusting hemostatic valve by Vygon (product code 1135.08; Vygon SA, Ecouen, France) with a male luer lock and just a short side outlet weighing only 3 grams, which fits perfectly to the Bard biopsy system, as its effective length when connected to the guiding needle measures exactly 3 cm. So the 7 cm guiding needle connected with the hemostatic valve can be handled with the inner cannula and the corresponding biopsy needle of the 10 cm device and the 10 cm guiding needle, as appropriate, with the 13 cm device, without any additional length compensation (Figure 3). Only the 13 cm guiding needle connected with the hemostatic valve needs a length compensation of 1 cm for use with the 17 cm inner cannula and 20 cm biopsy needle, for which the included depth stop is well-suited. The outer cannula of the guiding needle is connected with the hemostatic valve and flushed with saline solution over its side outlet, and the inner cannula of the one size larger coaxial needle is inserted.

The combination of materials as mentioned above reduces the maximal working length of the guiding cannula from 17 cm to 13 cm and increases material consumption, but it enables coaxial biopsy without opening the guiding needle to the atmosphere. Lung biopsies at our institution are performed both with 19/20-gauge and 17/18-gauge coaxial needles. Lesions with increased risk factors for systemic air embolism are biopsied with a 19/20-gauge device.

**Figure 4:**
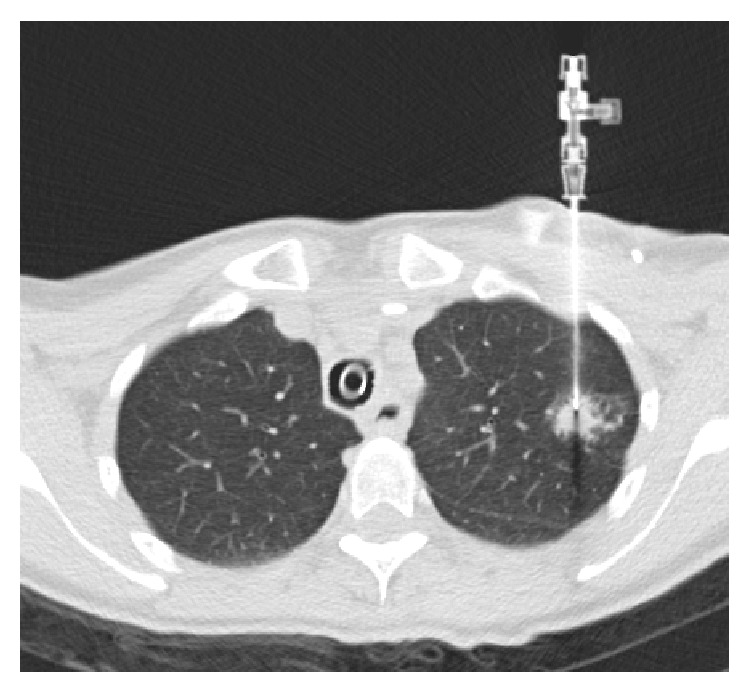
CT image during lung biopsy with guiding needle combined with hemostatic valve as described (under intubation anaesthesia at the explicit request of the patient, with venous port system visible in the edge region).

Based on our experience the combination of both products is quite feasible (Figure 4). Furthermore, the combined device makes the biopsy procedure more comfortable for the performing radiologist. Using a hemostatic valve for coaxial biopsy of lung lesions opens up the possibility to eliminate one risk factor for air embolism due to percutaneous lung biopsy.

## 5. Discussion

Risk factors for air embolism due to percutaneous lung biopsy can be subdivided into the following categories: patient factors, lesion factors, and technical factors. Since patient factors such as patient compliance, lung emphysema, or coughing during the procedure and lesion factors, in general, may be influenced only to a very certain degree, if at all, technical factors remain the key for resolving or at least reducing the problem.

Considering possible risk factors for air embolism due to percutaneous lung biopsy needs to begin with a look at the underlying mechanism. One mechanism is the creation of a fistula between air-containing space and a pulmonary blood vessel or vein with the biopsy needle. A second mechanism is opening the outer cannula of a coaxial biopsy needle to the atmosphere. The third mechanism occurs with one of the aforementioned mechanisms, but with transcapillary passage of air from a pulmonary artery, in the way of paradoxical air embolism [48]. The third mechanism of transcapillary route of embolism, however, occurs primarily, if not exclusively in other procedures where large amounts of gas or microbubbles caused by electrosurgical vapours enter the right heart, as, for example, in the case of so-called high-risk procedures in the context of central venous access, hysteroscopic surgery, or gastrointestinal endoscopy [49–51].

A fistula between air-containing space of the lung and a pulmonary vein causes an air embolism only in the presence of an additional positive pressure difference, in other words, when atmospheric pressure exceeds pulmonary venous pressure. This is the case, for example, in the event of coughing or deep breathing during and rarely even after the biopsy procedure.

From the facts listed so far, it is evident that needle path through ventilated lung, biopsy of small lesions, biopsy of a cavitary lesion, coughing during the procedure, positive pressure ventilation, or biopsy of a more vulnerable lesion as due to, for example, vasculitis obviously and without doubt can be considered risk factors for air embolism. From these, however, only the factor positive pressure ventilation can be regarded as essentially influenceable or avoidable.

Creation or, better said, widening of a fistula otherwise could be facilitated by other factors such as greater respiratory motion of the corresponding lung areas during lesion puncture with enlarging of the needle tract, which is more likely during procedures in the lower lobes. Here, greater respiratory motion may also make the procedure more difficult and necessitate a greater number of redirections of the needle [4]. Concerning the abovementioned correlations, the question of what the role of patient position during lung biopsy is up to this point, however, remains unanswered. In particular, whether prone position might be considered a risk factor for air embolism is a question of debate [3, 4] and for this reason was the subject of our literature review.

With respect to CT-guided lung biopsy in general, there is no overall strategy or recommendation concerning patient positioning. In many situations, the selected access route to the lesion with regard to the shortest needle track through the lung, avoiding fissures, large vessels, or bullae seems to be beyond question and determines the appropriate patient positioning. The latter allows various options for the radiologist. Many radiologists prefer, whenever possible, the supine or prone position to the lateral one as it is generally considered more stable and consistent [52]. A prone or supine position has also been recommended for the prevention of air embolism in the “Guidelines for radiologically guided lung biopsy” of the British Thoracic Society but significantly without any reasonable justification for this [53]. However, a lesion that can easily be reached from the back in a prone position usually can be reached from the back in a lateral position as well. In Rozenblit's investigation in this regard, he stated that in most cases (78%) transthoracic needle biopsy in an ipsilateral dependent position would be no more technically difficult than a routine chest biopsy [54]. Concerning preventive risk management when intrapulmonary hemorrhage with hemoptysis occurs, generally speaking the ipsilateral positioning during biopsy is the optimal position to avoid transbronchial spillage of blood in lung areas far apart from the lesion. So, all in all there is no compelling reason why prone positioning of the patient during a lung biopsy might be essential in general.

The two publications that have investigated most cases of air embolism due to CT-guided lung biopsy are those of Freund et al. [3] and Ishii et al. [4] with these also presenting the most compelling evidence. Both studies present level III evidence by using the Oxford Centre for Evidence-Based Medicine 2011 guidelines [55].

In the retrospective observational study of Freund, the publication with most of the cases of air embolism documented by far ever in a single institution, 19 of 23 (82,6%) cases of air embolism occurred in prone position and prone position turned out to be a significant risk factor for air embolism in both the univariate and the multivariate analysis. Consequently, Freund recommends avoiding prone position in CT-guided lung biopsy. Additional risk factors in Freund's analysis were the depth of the needle in the lesion, endotracheal anaesthesia, and the location of the lesion above the level of the left atrium. In the latter, the pulmonary venous pressure is reduced and the likelihood of a positive pressure difference between air-containing space of the lung and a pulmonary vein is increased.

The conclusions of the recently published Japanese multicenter case-control study of Ishii et al. [4] stand in contrast to those of Freund. Although even here most cases of air embolism, seven out of ten, occurred in prone position, in the univariate and multivariate analysis of Ishii, patient positioning was not significantly associated with the occurrence of air embolism. However, a closer look at the investigation reveals two points of the analysis and interpretation which are at least disputable.

First, a detailed look at the data shows that the patient positioning factors compared were “supine” versus “prone and lateral” and not versus prone exclusively, which does not allow a clear statement regarding the inquiry of prone position being a possible risk factor.

Furthermore, in the point of view of Ishii, the true risk factor explaining his presented data was “location of the lesion in the lower lobe” and not prone position itself, as most of patients with a lesion in the lower lobe were positioned in prone. Ishii stated that location in the lower lobe would be an “inevitable” risk factor for air embolism, because of greater vessels in the lower parts of the lung and greater respiratory motion of the lower parts of the lung, the latter one resulting in enlargement of the needle tract and a more difficult procedure requiring a greater number of redirections of the needle. This view, however, does not consider that part of this, namely, a greater respiratory motion of the lung, is an influenceable risk factor that can widely or completely be eliminated if it is possible to carry out the biopsy from the back not in prone, but in ipsilateral dependent position. In lateral patient positioning the ipsilateral lung is compressed, its volume is reduced, and motions of the ipsilateral hemithorax and diaphragm are reduced resulting in an overall hypoinflation of the dependent lung, a fact well known from both lung biopsy and also, for example, adrenal biopsy [54, 56]. So the risk factors “greater vessels” and “greater respiratory motion” in the lower lobes can be, but do not necessarily have to be connected with each other. In other words, the probably minor risk factor “location in the lower lobe” due to greater vessels in the lower parts of the lung becomes a probably major one and is, respectively, potentiated by the risk factor “prone position” promoting greater respiratory motion of the lower parts of the lung, which can be minimised by using ipsilateral dependent position. This would also automatically fulfil the requirement of Freund concerning avoidance of the factor “lesion location above the left atrium” in lateral patient position.

Prone position also might be a risk factor for air embolism or better said for the clinical manifestation of air embolism for the following reasons.

Anatomically speaking, in a supine or lateral patient position air might be more likely to stay in a pocket in the left heart or ascending aorta before it gets further into the systemic circulation than air in a prone position. But, this is of course speculative.

Another similar explanation could be that when performing a CT-guided lung biopsy in a prone position the patient often is turned back to the supine position before a control-scan of the entire heart and ascending aorta has been conducted to rule out air embolism. This rotational manoeuvre of 180 degrees can cause the clinical manifestation of air embolism and is normally reduced to 90 degrees in a biopsy in lateral position and most often is totally prevented in a biopsy in supine position.

The papers of Freund and Ishii, supported by the data of our systematic literature review, provide important indications that lung biopsy of a lesion in the lower parts of the lung in prone position contains a combination of risk factors for the development of systemic air embolism based on the two single factors greater vessel calibre and greater respiratory motion of the lower parts of the lung with prone position being the influenceable and usually commonly avoidable risk factor.

One can statistically evaluate the data of our literature review with, on the one hand, far more than half of all air embolisms having occurred in prone position. However, on the other hand, most publications dealing only with one-case reports without information about data of the whole collective of biopsied patients are, quite naturally, without significance. Nevertheless we believe that our data has clinical relevance.

The practical conclusions from our point of view, however, are a bit different from those of Freund, who recommends that “whenever possible patients should be positioned on the back in such a way that the tumour is lower than the left atrium.” We believe this requirement to be impractical. Beyond the general aspects of percutaneous lung biopsy, such as avoiding pleural fissures and avoiding long needle track through lung parenchyma, the two risk factors “lesion above the level of the left atrium” and “greater respiratory motion,” in our opinion, have the following consequence: dorsal located lesions should be biopsied not in prone, but in ipsilateral dependent position. The question of whether all ventral located lesions should also be biopsied in an ipsilateral dependent position or in supine position is a bit more difficult to answer. A biopsy of a ventral lesion from an anterior or ventral access in an ipsilateral dependent position seems hardly feasible, at least in women and presumably also in men, due to compressed soft tissue of chest muscles, breast parenchyma, and subcutaneous fat in this position. From our point of view, ventral lesions should be biopsied in supine position for technical feasibility, ignoring the risk-factor “lesion above the level of the left atrium.” Lateral lesions can be biopsied in supine position or from the back in ipsilateral dependent position.

The use of a hemostatic valve for coaxial biopsy of lung lesions as described in our technical note is a quite simple option to securely prevent opening the outer cannula to the atmosphere and thus eliminate one risk factor for air embolism due to percutaneous lung biopsy.

Not a risk factor for air embolism itself, but impacting its clinical consequences, is the correct way of handling the patient immediately after conducting the biopsy. In most current practice, the postprocedure CT scan only includes the target area of biopsy for the observation of pneumothorax and biopsy-tract haemorrhage. Early detection of air embolism in the left atrium or left ventricle, however, can prevent air embolism in the systemic circulation resulting from this. For this purpose, a CT scan of the whole aortocardiac region with the patient in an unaltered position as related to biopsy position should be performed to find any evidence of air embolism. If air embolism hereby can be ruled out, the patient position can be altered. If air embolism is recognised, any alteration of the patient position should be considered thoroughly to retain the air in the relative safe position of the left atrium or left ventricle. Repositioning the patient in the situation when air embolism has been detected might be a very critical point and should be decided on a case-by-case basis, perhaps only after transcatheter or even percutaneous-transcardial air-aspiration. This general advice has been given previously by several other investigators [23–25] but since now has not been established sufficiently in daily practice. The clearest example of this is the practice in our clinic, as it has been for many years before the event of the aforementioned case.

In conclusion, there is low-level evidence that possibly explains why prone position is a risk factor for the development and/or clinical manifestation of systemic air embolism due to CT-guided lung biopsy of lesions in the lower parts of the lung. Prone position, therefore, should be avoided for biopsy of lesions in the lower lobes or lower parts of the lung and replaced by ipsilateral dependent positioning of the patient, especially in situations where other risk factors for air embolism, such as small or cavitary lesions or needle path through longer distances of ventilated lung, are present.

For biopsies of small or cavitary lesions in coaxial technique, using a hemostatic valve is strongly recommended in general.

Furthermore, we recommend a control-scan of the entire thorax immediately after lung biopsy in an unaltered patient position before any repositioning of the patient, as has been previously recommended by several other investigators.

Recommendations for CT-guided lung biopsy should be propagated among radiologists in national or international guidelines.

## Figures and Tables

**Table 1:** Analysed publications with information about patient positioning.

Authors [references]	Number of cases	Patient positioning					
		P	S	L	R	Lat	N
36 [5, 6, 10–22, 25, 26, 28–45 ∗]	1	16	11	6	3	0	(0)
Kuo et al. [23]	2	0	2	0	0	0	(0)
Ibukuro et al. [24]	3	1	2	0	0	0	(0)
Hare et al. [2]	4	3	1	0	0	0	(0)
Um et al. [27]	4	1	2	0	0	0	(1)
Ishii et al. [4]	10	7	2	0	0	1	(0)
Freund et al. [3]	23	19	0	0	0	0	(4)

42	82	47	20	6	3	1	(5)

P: prone, S: supine, L: left-lateral, R: right-lateral, Lat: lateral but not specified, N: not mentioned.

∗: own case report.
